# Psychometric properties of the affect integration inventory – short form in a sample of patients with personality disorder

**DOI:** 10.3389/fpsyg.2023.1191752

**Published:** 2023-10-31

**Authors:** Christina Frederiksen, Gry Kjaersdam Telléus, Ole André Solbakken

**Affiliations:** ^1^Psychiatry, Aalborg University Hospital, Aalborg, Denmark; ^2^Department of Communication and Psychology, Aalborg University, Aalborg, Denmark; ^3^Department of Psychology, University of Oslo, Oslo, Norway

**Keywords:** affect integration, affect integration inventory, affect integration inventory – short form, affect consciousness, personality disorder, emotional dysfunction

## Abstract

**Introduction:**

Affect integration comprises the capacity to access and utilize the motivational and signal properties of affects. This capacity is essential for personal adjustment, mental health, and well-being. Affect integration is commonly operationalized through the Affect Integration Inventory. This study examines the psychometric properties of a short-form (AII-SF-42) of the instrument in a sample of patients with personality disorders (*n* = 87).

**Methods:**

Analyses of internal-consistency reliability, along with standardized mean differences-, and associations between short- and long-forms are reported. Internal structure was assessed by confirmatory factor analyses and external criterion validity was addressed by tests of associations between the AII-SF-42-scale scores and measures of alexithymia, symptom distress, interpersonal problems and level of personality dysfunction.

**Results:**

The study demonstrated satisfactory reliability and validity for scores derived from the AII-SF-42, including acceptable internal consistency and strong correspondence with long-form scores, a consistent factor structure organized according to discrete affects, and systematic patterns of convergent and discriminant associations with external measures.

**Conclusion:**

Taken together, the results of the study demonstrate that in clinical settings, including patients with personality disorders the AII-SF-42 is a valid and useful alternative to the full-length version of the instrument.

## 1. Introduction

Emotional dysfunction is a major component in the conceptualization of personality disorder (PD) (American Psychiatric Association, 2013) and several studies have established a close connection between dysfunctional management of emotions and PD-psychopathology (Solbakken et al., 2011a; Johansen et al., 2013, 2016; Normann-Eide et al., 2013; Frederiksen et al., 2021a,b). However, little agreement has been reached on how the concept of emotional dysfunction is best addressed empirically, especially in terms of operationalized criteria for measuring variation across emotion states or discrete affects (Berking and Wupperman, 2012). One promising conceptualization that has received empirical support is Affect Integration (AI).

AI is defined as the capacity to utilize one’s affects for adaptive purposes. The concept refers to those processes that influence the availability of affects for motivating, guiding, and informing individuals in their transactions with their surroundings (Monsen and Monsen, 1999; Solbakken et al., 2011a). The capacity to integrate affects into motivation, cognition, communication, and behavior is postulated as a hallmark of personal adjustment and well-being by ensuring appropriate responses to the varying circumstances facing the individual. Accordingly, deficits in the capacity for affect integration may result in severe breakdowns in cognitive, emotional and relational functioning (Solbakken et al., 2011a, 2017; Frederiksen et al., 2021a).

The AI-construct encompasses both the capacity for accessing and utilizing the adaptive properties of discrete affects for personal adjustment and the capacity for tolerating and regulating affects (Monsen et al., 1996; Monsen and Monsen, 1999; Solbakken et al., 2011b). Other theoretical concepts also address the relationship between affect activation and its impact on thoughts and behavior, such as alexithymia (Lesser, 1981; Bagby et al., 1994), mentalized affectivity (Fonagy et al., 2002; Jurist, 2005), emotion regulation (Gross and Thompson, 2007), levels of emotional awareness (Lane et al., 1990), emotional understanding (Hellwig et al., 2020), and emotional intelligence (Salovey and Mayer, 1990). What sets the AI-construct apart from these concepts is a particular emphasis on the differentiation of discrete affects/emotion states and their unique informational and motivational impacts (Choi-Kain and Gunderson, 2008; Solbakken et al., 2011b).

AI was first operationalized and assessed by the observer-based Affect Consciousness Interview (ACI: Monsen et al., 2008) and later by the self-rated Affect Integration Inventory (AII: Solbakken et al., 2017; Frederiksen et al., 2022). Both instruments include the assessment of several different affects, which are seen as biologically and evolutionarily founded responses. It is posited that affect processes are highly idiosyncratic and, due to the complex influences of the individual’s cultural and unique developmental history, become organized and automatized as prototypical patterns (or scripts) of experiencing, understanding, and expressing affective reactions (Tomkins, 2008a, 2008b; Solbakken et al., 2011b). In line with this, the ACI and the AII operationalize the individual’s capacity for functional affect integration in terms of the adaptiveness of the individual’s experience and expression of discrete affects.

Various studies have identified and demonstrated the usefulness of this operationalization (Monsen et al., 1996; Monsen and Monsen, 1999; Gude et al., 2001; Waller and Scheidt, 2006; Choi-Kain and Gunderson, 2008; Lech et al., 2008; Solbakken et al., 2011a, 2012, 2017; Johansen et al., 2013; Normann-Eide et al., 2013; Falkenström et al., 2014; Taarvig et al., 2015; Taarvig and Solbakken, 2018; Frederiksen et al., 2021a,b).

Importantly, the observer-rated ACI is time-consuming to administer, which somewhat limits its applicability. Likewise, the complete AII, originally with 112 (and in its revised, extended form 137) items, is in many cases too lengthy to apply, e.g., in studies that include several questionnaires in larger test-batteries. For that reason, the need for a brief version of the AII was raised and a 42-item short-form was developed and validated in a non-clinical context (Solbakken and Monsen, 2021).

The present study examines the usefulness of AII-SF-42 for measuring AI in a sample of patients with PD and is the first of its kind on this short form of the AII in a clinical context. The study includes an examination of differences in magnitude of scores derived from the short- and the longer 112-item form of the AII, along with estimates of internal consistency reliability, examination of internal structure of scores through confirmatory factor analysis (CFA), and tests of convergent and discriminant validity. The utility of systematically distinguishing discrete affects or emotion states was tested by examining relationships between the integration of discrete affects and specific theoretically hypothesized patterns of interpersonal problems.

However, when dealing with the internal domain study, internal consistency reliability and the correspondence between short- and long forms of AII-scale scores was analyzed. This correspondence was expected to be high, as evidenced by very strong or near perfect correlations between the two versions and small differences in absolute levels of scores on corresponding long- and short-form scales.

Afterwards the internal structure of scores was tested. According to the theoretical underpinnings of the AII and the AI-construct, scores can be organized in terms of four competing factor models which can be tested against each other. The first is a general AI-factor model, suggesting that that AII-SF-42 scores are best represented by one overarching general factor. This model is consistent with concepts such as level of emotional awareness (LEAS) and emotional intelligence and implies that the assessment of variation between affects is of little importance and that variation in affective functioning is more general in nature. The second is a differentiated integration of positive/negative affect model, suggesting that variation in AII-SF-42 scores is best represented by two separate, but related factors corresponding to capacity for integrating pleasant or positive affects on the one hand and unpleasant or negative affects on the other. Here too the variation in scores associated with discrete affects is considered of less importance, while differences between pleasant or positive and unpleasant or negative emotional states are considered central. The third model is a discrete affect model, which indicates that scores are best represented by nine related, but differentiated affect factors, indicating that integration of discrete affects constitute a fundamental organizing principle underlying the structure of the scores. The fourth model is a hybrid or bifactor model that combines a general factor that cuts across affects with a discrete affect model, in which both general affect integration and affect specific integration represent meaningful structural variation. In line with existing theory and research on the AI, it was hypothesized that the discrete affect model would be superior to the general and pleasant/unpleasant affect models, while the hybrid or bifactor model in turn would be superior to the discrete affect model.

In the external domain study central aspects of criterion related validity, more specifically concurrent, convergent, and discriminant validity, were examined by tests of associations with various external criterion measures. As a test of concurrent validity, associations with alexithymia were examined. In terms of convergent validity, associations with symptom distress, overall levels of interpersonal problems, and levels of personality functioning were examined. Finally, as a comprehensive test of convergent and discriminant validity, theoretically hypothesized patterns of relationships between the integration of discrete affects and specific types of interpersonal problems were examined.

AI and alexithymia share similar content domain as both address characterological capacities for experiencing and expressing emotions. Thus, in regard to the relationship between scores on the AII-SF-42 and alexithymia, measured by the Toronto Alexithymia Scale 20 (Bagby et al., 1994) strong negative associations between scores on all levels were expected. On subscale level a more differentiated pattern of associations is to be expected between scores on the AII-SF-42 and the three TAS-20 subscales; Difficulties Identifying Feelings, Difficulties Describing Feelings, and Externally Oriented Thinking. For Difficulties Identifying Feelings and Difficulties Describing Feelings a strong negative relationship with AII-SF-42 scores on all levels was expected. As Difficulties Identifying Feelings operationalizes difficulties in awareness and identification of emotions, the strongest association was expected to be with the AII-SF-42-scale Capacity for Experience. Conversely, Difficulties Describing Feelings operationalizes difficulties in communication of emotional experiences, thus, the strongest relationship was expected to be between this scale and the AII-SF-42-scale Capacity for Expression. Finally, Externally Oriented Thinking only shares limited conceptual overlap with the AI construct, thus only small or moderate associations between this Alexithymia-subscale and AII-SF-42 scores was expected.

It is a key assumption in the AI-model that high levels of AI protect the individual against psychopathology, aids in interpersonal adjustment, and ensures adaptive and flexible responses to strain and changes in the environment. Thus, a strong negative relationship between AII-SF-42 scores on all levels and the overall level of psychiatric symptoms (as measured by the General Severity Index (GSI) of The Symptom Checklist-90 Revised (SCL-90-R: Derogatis, 1994)) and with the overall level of interpersonal problems (as measured by the overall IIP-Global score) of The Inventory of Interpersonal Problems 64 (IIP-64: Horowitz et al., 2000) was expected. In line with previous studies (Monsen et al., 1996; Solbakken et al., 2011a; Frederiksen et al., 2021a), it was expected that the Capacity for Experience was more closely related to levels of symptom distress and interpersonal problems than the Capacity for Expression.

Similarly, problems with AI were expected to be closely related to the level of personality dysfunction. Prior research has revealed a close link between low levels of AI, and more pronounced personality dysfunction, especially in the areas of Identity Integration, Relational Functioning and Self-control (Frederiksen et al., 2021a). The present study was expected to replicate these findings. More specifically, we expected strong or moderate to strong relationships between the domains of Identity Integration, Relational Functioning and Self Control as measured by The Severity Indices of Personality Problems (SIPP-118: Andrea et al., 2007) and AI-scores on all levels, along with moderate relationships with the personality functioning domains of Responsibility and Social Concordance.

Finally, as a comprehensive test of patterns of convergent and discriminant validity, the relationships between scores on specific affect-scales from the AII-SF-42 and various systematically interrelated types of interpersonal problems as measured by the IIP-64 were examined. The interpersonal problem types operationalized in the IIP-64 is organized in a circular order constituting the interpersonal circumplex (Horowitz et al., 2000), therefore this measure is particularly well suited for exploring hypotheses about convergent and discriminant validity of scores from the AII-SF-42. The circular order of scales in the IIP-64 makes it possible to predict that the relationships between discrete affect scores on the AII-SF-42 and specific types of interpersonal problems will constitute distinct sinusoidal patterns of associations peaking in separate and theoretically expected octants (Solbakken et al., 2012, 2017). More specifically:

1. Problems with Tenderness, Sadness and Guilt (often interfering with the capacity for closeness, bonding, and attachment) are expected to have sinusoidal patterns of correlations peaking in the cold-detached octant.
2. Problems with Anger (often interfering with self-assertion and agency) are expected to have a sinusoidal pattern of correlations peaking in the non-assertive octant.
3. Problems with Jealousy (often interfering with trust and tolerance of interdependence on the other) are expected to have a sinusoidal pattern of correlation peaking in the vindictive octant.
4. Problems with Interest, Joy, Shame, and Fear (all of which often interfere with closeness/bonding as well as assertive behavior and combinations of the two) are expected to have sinusoidal patterns of correlations peaking in the socially inhibited/avoidant octant.

## 2. Methods

### 2.1. Procedures

Data for this study were collected cross-sectionally at two hospital-based outpatient units specialized in the treatment of PDs in the Psychiatric Health Care Services of the North Denmark Region. The treatment offered primarily consisted of weekly psychotherapy either in individual, group, or combined settings. The diagnostic status of the patients was assessed by the semi-structured Present State Examination (PSE: Committee and Kline, 2002) and structured clinical interview for DSM – IV axis II personality disorders (SCID-II: First et al., 1997). The interviews were conducted by experienced psychiatrists and psychologists who were trained in the use of the instruments. Final diagnostics were determined according to the Diagnostic and Statistical Manual of Mental Disorders, fifth edition (DSM-5: American Psychiatric Association, 2013). The online self-administered platform SurveyXact (Ramboll, Aarhus, Denmark) was used to collect self-reported data on symptom distress, interpersonal problems, affective- and personality functioning. Before entering the study, patients were informed that participation was voluntary, and that nonparticipation would have no consequences for their treatment in any way. The study was managed in accordance with the Helsinki Declaration and was approved by the Danish Data Protection Agency on 1 May 2014 (2019–017816). Written and oral information about the study was provided before inclusion. Due to the nature of the study, no further approval was needed from the Danish National Committee on Biomedical Research Ethics.

### 2.2. Participants

Patients referred to treatment for PD at one of the outpatient units, meeting the inclusion criteria of a diagnosis of PD according to the DSM-5 (American Psychiatric Association, 2013), aged 18 years or above, being literate in Danish, and providing an informed written consent to participate, were recruited to the study. Patients with comorbid psychotic disorder and bipolar I disorder were treated elsewhere and for that reason excluded from the study, as was patients with developmental disorders (e.g., Asperger’s disorder), or a diagnosis of drug or alcohol dependence potentially interfering with the outcome of treatment.

### 2.3. Instruments

The Affect Integration Inventory Short and full Forms (Solbakken et al., 2017; Solbakken and Monsen, 2021): The AII is a self-reported instrument for the assessment of affect integration. It comprises 112 statements about perception of awareness, tolerance, and expressions of nine discrete affects: (1) Interest, (2) Joy, (3) Fear, (4) Anger, (5) Shame, (6) Sadness, (7) Jealousy, (8) Guilt, and (9) Tenderness. Eighty-two of the statements are indicators of the capacity for adaptive experience, while 30 are indicators of capacity for adaptive expression. All items are rated on a 10-point Likert scale ranging from does not fit at all (0) to fits perfectly (9). Higher scores indicate higher levels of functioning. Scores from the AII can be calculated on different levels: (1) a mean overall score (Global AI), (2) a mean score on the capacity for experience across affects (Experience) or a mean score on the capacity of expression across affects (Expression), and (3) mean scores for integration of each of the discrete affects (e.g., integration of Joy, Sadness etc.).

The AII- SF-42 was based on the original AII and developed according to a pre-determined four-step procedure which has been described more thoroughly by Solbakken and Monsen (2021). In the AII-SF-42 the differentiation between the nine affects has been maintained, along with the conceptual distinction between indicators of Experience and Expression within each affect category. In the present study, all 112 AII-items from the full form were administered once and separate scores were in turn computed for long- and short-forms separately. Cronbach’s alphas for the full 112-item version of the AII in the study were 0.94 for Global AI, 0.91 for Experience, and 0.91 for Expression 0.70 for Sadness, 0.78 for Anger, 0.86 for Tenderness, 0.73 for Guilt, 0.78 for Fear, 0.77 for Shame, 0.81 for Interest, 0.84 for Joy, 0.92 for Jealousy.

The Toronto Alexithymia Scale 20 (TAS-20: Bagby et al., 1994): The TAS-20 is a 20-item self-reported scale for measuring alexithymia. Each item is rated on a five-point Likert scale ranging from strongly disagree (1) to strongly agree (5). The TAS-20 comprises three subscales: The Difficulty Identifying Feelings subscale (seven items); the Difficulty Describing Feelings subscale (five items) and the Externally Oriented Thinking subscale (eight items). The TAS-20 is widely studied and one of the most commonly used evaluations of alexithymia. The Cronbach’s alpha values for the sample were 0.85 for the Global Alexithymia scores, 0.81 for Difficulty Identifying Feelings, 0.66 for Difficulty Describing Feelings, and 0.62 for Externally Oriented Thinking.

The Symptom Checklist-90, Revised (SCL-90 R: Derogatis, 1994): The SCL-90-R is a self-reported scale designed to assess psychopathological symptoms. The scale includes 90 items addressing the intensity of symptoms during the last 7 days. Items are rated on a 5-point Likert scale ranging from not at all (0) to very much (4). The global severity index (GSI) is calculated as an average across all 90 items and serves as an indicator of the current level of general distress. Cronbach’s alpha value for the GSI was 0.95.

Inventory of Interpersonal Problems 64 Circumplex Version (IIP-64: Horowitz et al., 2000): The IIP-64 is a self-reported scale assessing the level of relational/interpersonal problems. The items are rated on a 5-point Likert scale ranging from not at all (0) to very much (4). The IIP-64 yields one overall score (IIP-Global) and eight octant subscale scores. The sub-scales scores are organized in a circular order thereby constituting an interpersonal circumplex (Horowitz et al., 2000). Whereas the IIP-Global measures the general level of interpersonal problems, each of the eight octant scores assess specific and systematically interrelated types of interpersonal problems with being: Domineering, Vindictive, Cold, Socially Inhibited, Nonassertive, Overly Accommodating, Self-sacrificing, or Intrusive. Studies have linked the IIP-Global to symptom severity and negative affectivity (Tracey et al., 1996), and the circumplex structure has demonstrated good construct validity in terms of fit and patterns of convergent-discriminant associations with external correlates (Monsen et al., 2006). Cronbach’s alpha estimates were 0.90 for IIP-Global, 0.76 for Domineering, 0.72 for Vindictive, 0.80 for Cold, 0.81 for Socially Inhibited, 0.87 for Nonassertive, 0.73 for Overly Accommodating, 0.73 for Self-sacrificing, and 0.73 for Intrusive.

The Severity Indices of Personality Problems (SIPP-118: Andrea et al., 2007): The SIPP-118 is a self-reported questionnaire intended to measure centrale aspects of personality dysfunction. The assessment of personality dysfunction in the SIPP-118 links to the diagnostic approach presented in the Alternative Model of Personality Disorder presented in section 3 of the DSM-5 and the upcoming ICD-11 (Bender et al., 2011; Tyrer et al., 2019). The questionnaire consists of 118 items that are rated on a 4-point Likert scale ranging from “I fully agree” to “I fully disagree.” Higher scores equal more adaptive functioning. The 118 items can be converted into 16 facets and organized into five higher-order domains: (1) The Identity Integration Domain; (2) The Relational Functioning Domain; (3) The Self-Control Domain; (4) The Social Concordance Domain; and (5) The Responsibility Domain. Previous studies on the SIPP-118 have not been unambiguous (Bastiaansen et al., 2013). However, three studies have reported good psychometric properties, including cross-national consistency (Verheul et al., 2008; Arnevik et al., 2009; Feenstra et al., 2011). The Cronbach’s alpha values for the sample were 0.88 for the Self-control Domain, 0.84 for the Identity Integration Domain, 0.69 for the Responsibility Domain, 0.79 for the Relational Functioning Domain and 0.84 for the Social Concordance Domain.

### 2.4. Statistical analyses

Statistical analyses were done using the SPSS version 26 and its AMOS module for structural equation modeling. Descriptive statistics were computed for the relevant study variables. Internal consistency reliability was estimated using Cronbach’s alpha. For testing the correspondence of short- and long-form versions of the AII, the standardized mean differences of corresponding scale scores were computed, along with bivariate Pearson’s r correlation coefficients.

The factor structure of AII-SF-42 scores was assessed through Confirmatory Factor Analyses (CFA) with Structural Equation Modeling (SEM). Since CFA is sensitive to the ratio of participants to the number of items and estimated parameters, a set of three representative indicators for each affect were produced (Furr, 2011). For scales consisting of four items (Joy, Tenderness, and Jealousy), a mean score of two items randomly selected to sample the complete construct domain of the affect in question was computed and used as an indicator, while the two remaining items were entered directly into the analyses. For scales consisting of five items (all remaining affects), a mean score of two sets of two items each randomly selected to sample the complete construct domain of the affect in question was computed and used as separate indicators, while the one remaining item was entered directly into the analyses. Thus, it was ensured that all AII-SF-42 items were represented once and only once within the indicator set and resulting analyses. All SEM-computations were done with maximum likelihood estimation. Competing theoretical factor models were compared using common comparative and absolute fit indexes, i.e., chi-squared, AIC, BIC, RMSEA, IFI, and CFI. Non-nested models were formally selected based on AIC/BIC, while nested models were selected based on the chi-squared. Following (Brown, 2015) recommendation, the following values were considered acceptable model fit; a root mean square error of approximation (RMSEA) close to or below.08, a comparative fit index (CFI) and an incremental fit index (IFI) close to or greater than 0.90. Finally, modification indices were used to identify possible adjustments and improvements to the best fitting model.

Associations with external criteria were analyzed using bivariate Pearson’s r correlations. Z-tests were conducted to assess the statistical significance of differences in correlation magnitude for demonstrating convergent and discriminant validity. Correlation magnitudes were interpreted according to (Cohen, 1988) classifications of effects, i.e., correlation coefficients in the order of 0.10 are small, those at approximately 0.24 are medium, and those at approximately 0.37 are large. Color charts were included in correlation tables to ease interpretation. Sinusoidal structure and fit of association patterns of integration of discrete affect scores with interpersonal circumplex/IIP-subscales was assessed using Gurtman and Balakrishnan’s (1998) structural summary method and its corresponding Goodness of Fit-index (GoF).

## 3. Results

The sample consisted of 87 participants of which most were female (85%). Mean age was 31.7 years (SD = 9.5). On average the participants were diagnosed with 1.4 PD-diagnoses meaning that several of the participants were diagnosed with more than one PD-diagnosis. The most common diagnosis was avoidant PD (41%), followed by borderline PD (34.5%), and mixed PD (17.2%). A smaller subset of the sample was diagnosed with either, obsessive-compulsive PD, Narcissistic PD or Paranoid PD (6.9%). Also, 31% suffered from comorbid mood disorder, 24.1% from anxiety disorder, 4.6% from substance abuse, 3.4% from eating disorder and 4.6% from behavioral disorder.

In Table 1 descriptive statistics and internal consistency estimates for the AII-SF-42, as well as standardized mean differences between the long and the short forms of the AII are displayed. The median alpha value was.78, with a range from.58 to.89. Thus, Cronbach’s alpha values were generally high and suggesting fair to excellent internal consistency. The standardized mean differences between the corresponding scores of the short- and full-form of the AII were small to negligible with a median of 0.15, suggesting very high coherence between scores from the two versions of the instrument. Accordingly, correlations between corresponding full- and short-form scale scores ranged between.80–0.98 with a median of.91 again suggesting very strong to excellent agreement between corresponding scores.

**Table 1:** Descriptive statistics and reliability estimates for AII-SF-42 scores, along with standardized mean differences from and correlations with corresponding AII-full-scale scores.

	Minimum	Maximum	Mean	Standard deviation	α	d	*r*
Global AI	1.30	6.81	3.60	1.19	0.89	−0.15	0.96
Experience	1.33	6.25	3.56	1.15	0.78	−0.13	0.94
Expression	0.63	8.05	3.65	1.50	0.86	−0.09	0.98
Interest	0.60	9.00	4.11	2.16	0.79	−0.16	0.91
Joy	0.00	9.00	4.56	2.17	0.59	0.46	0.80
Fear	0.00	7.67	2.46	1.87	0.58	−0.37	0.85
Anger	0.00	8.00	2.84	1.82	0.70	−0.31	0.92
Shame	0.00	9.00	2.83	1.87	0.74	−0.17	0.90
Sadness	0.00	8.00	2.99	1.79	0.59	−0.09	0.82
Jealousy	0.00	9.00	3.95	2.85	0.85	−0.02	0.95
Guilt	0.40	9.00	4.13	1.90	0.70	−0.15	0.91
Tenderness	1.00	9.00	5.23	2.11	0.64	0.31	0.94

α = Cronbach’s alpha, d = Standardized Mean Difference between long form and short form raw scores using pooled SDs across short- and full form scales, *r* = correlations between corresponding short-form and long-form scales.

Table 2 displays the results of the CFAs. As can be seen, the results show that among the competing models the hybrid or bifactor Discrete Affects and General AI Bifactor Model was the most apt in terms of fit followed by the Discrete Affects Model, Positive and Negative Affect Model, and finally the General AI Model. The best fitting model was acceptable in terms of RMSEA and IFI, but not in terms of the CFI. Accordingly, modification indices suggested five theoretically feasible adjustments to the model (i.e., the addition of five cross-loadings), the addition of which improved fit across all relevant indexes, including bringing the CFI into the acceptable range. Graphic presentations of the various models can be found in the supplementary materials.

**Table 2:** Comparison of competing structural models as tested by CFA.

Model	df	χ2	AIC	BIC	RMSEA	IFI	CFI
A	324	928,979	1,079,979	1,169,186	0,147	0,416	0,401
B	323	906,725	1,070,725	1,149,848	0,145	0,437	0,422
C	288	437,157	671,157	784,122	0,078	0,861	0,852
D	261	370,410	658,407	797,442	0,070	0,900	0,892
E	257	320,271	616,271	759,167	0,054	0,943	0,937

df = degrees of freedom, χ2 = chi squared, AIC = Aikake’s Information Criterion, BIC = Bayesian Information Criterion, RMSEA = Root Mean Square Error of Approximation, IFI = Incremental Fit Index, CFI = Comparative Fit Index, A = General AI Model, B = Positive and Negative Affect Model, C = Discrete Affects Model, D = Discrete Affects and General AI Bifactor Model, E = Discrete Affects and General AI Bifactor Model with Adjustments.

The correlations between the AII-SF-42 scores and scores for alexithymia, general symptom distress and overall level of interpersonal problems are presented in Table 3. Overall, results agreed with our hypotheses, indicating concurrent, convergent, and discriminant validity of AII-SF-42 scores. As expected, the correlations between the AII-SF-42 scores and TAS-20 scales were negative and generally strong or moderate to strong. On a subscale level, a differentiated pattern of correlations was found, including weaker correlations between AII-SF-42 scores and the TAS-External Oriented Thinking subscale than between AII-SF-42 scores and the other TAS scales, as evidenced by the detection of only small and moderate correlations with this subscale. Furthermore, it was hypothesized that Difficulty Identifying Feelings would be more strongly associated with Experience than with Expression and that Difficulty Describing Feelings would be more strongly associated with Expression than with Experience. Results showed that, in absolute terms, this was the case. However, in tests of the significance of differences in correlation magnitude none of these were statistically significant [Experience vs. Expression for TAS-DDF: *z* = 1.47, *p* = 09 (one-tailed), Experience vs. Expression for TAS-DIF: *z* = 0.90, *p* = 0.19 (one-tailed)].

**Table 3:** Colour-chart of correlations between AI-SF-42-scores and alexithymia-, general symptom severity- and overall interpersonal problem scores.

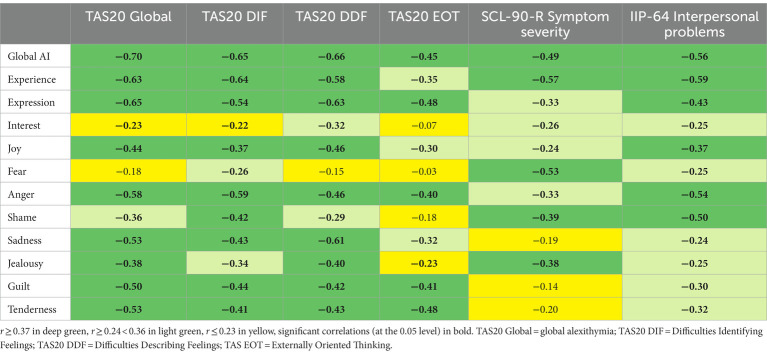

Analyses of the correlations between symptom severity (SCL-90R, GSI), general interpersonal problems (IIP-64, IIP-Global) and the higher order AII-SF-42 scales (Global AI, Experience, and Expression) revealed strong and moderate to strong negative associations as expected. Also, the results confirmed the hypothesis of a closer relationship between the level of symptom distress and interpersonal problems on the one hand and the capacity for experience on the other when compared to the capacity for expressing and communicating ones affects. A test for significance of differences in correlation magnitude demonstrated that these were statistically significant [Experience vs. Expression for GSI: *z* = 3.17, *p* = 0.002* (one-tailed), Experience vs. Expression for IIP-Global: *z* = 2.19, *p* = 0.014* (one-tailed)].

In Table 4, the correlations between the AII-SF-42 scores and the SIPP-118 domains are shown. Strong and moderate associations were expected between the AII-SF-42 scores and the SIPP-118 domains of Identity Integration, Relational Functioning and Self Control. Identity Integration was strongly or moderately correlated with all AII-SF-42-scales. Relational Functioning was strongly or moderately correlated with all AII-SF-42-scales except for integration of Fear. Self-control was strongly associated with all AI-scales except for integration of Fear, Interest, and Joy. Additionally, more modest associations were expected and obtained with the SIPP-118 domains of Social Concordance and Responsibility. With about half of the correlations being strong or moderate, the domain of Social Concordance was more closely related to the AII-SF-42 scales than the Responsibility domain, for which only three correlations were of moderate strength and the rest small or insignificant.

**Table 4:** Colour-chart of correlations with SIPP-118 personality functioning domains.

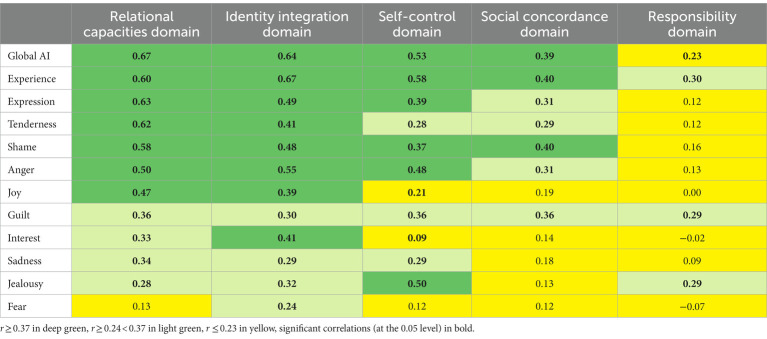

**Figure 1:**
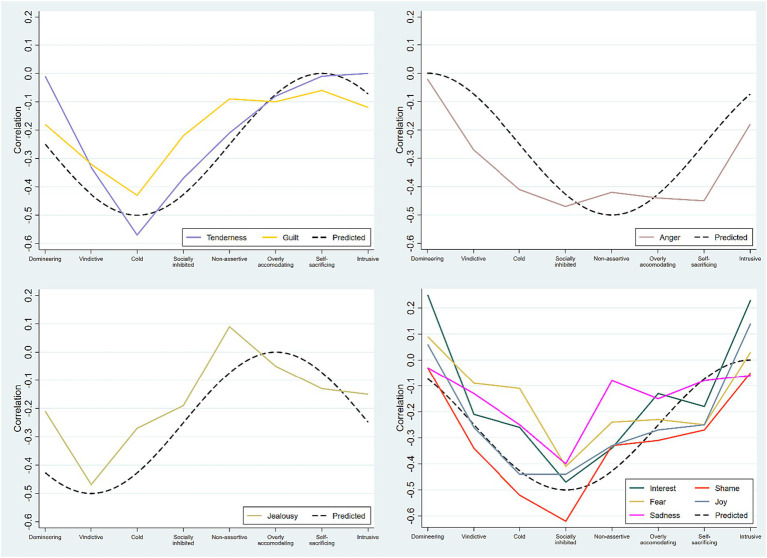
Patterns of relationships between the discrete affects and specific types of interpersonal problems. The predicted patterns are shown as black dashed lines. Note. Predicted and obtained patterns of correlations between discrete affect scores and the octant scales of the IIP-64. Upper left panel: The integration of Tenderness and Guilt both peaked in the predicted Cold/Detached octant. Upper right panel: The integration of Anger was more broadly associated with the Cold/Detached, Socially inhibited, Non-assertive, Overly accommodating, and Self-sacrificing octants. Lower left panel: The integration of Jealousy peaked in the predicted Vindictive octant. Lower right panel: The integration of Interest, Fear, Sadness, Shame, and Joy all peaked in the predicted Socially inhibited octant. All correlation patterns demonstrated high GoF with optimal cosine curve functions peaking in respective hypothesized octants.

Finally, in Figure 1 the hypothesized and observed patterns of associations between AII-SF-42 scores for integration of discrete affects and specific types of interpersonal problems are presented. As can be seen, results were generally consistent with our predictions. Problems with Jealousy had a sinusoidal correlation pattern peaking in the vindictive (BC) octant. GoF was high (0.91) with an optimal cosine curve function peaking in BC. Problems with Tenderness and Guilt had sinusoidal correlation patterns peaking in the cold-detached (DE) octant. Both correlation patterns had high Goodness of Fit (GoF; Tenderness = 0.94, Guilt = 0.95) with an optimal cosine curve function peaking in DE. Problems with Interest, Shame, Fear, Joy, and Sadness all had sinusoidal correlation patterns peaking in the socially inhibited (FG) octant. GoF was high (Interest = 0.87, Shame = 0.96, Fear = 0.88, Sadness = 0.93, Joy = 0.87) with an optimal cosine curve function peaking in FG. Problems with Anger was more broadly associated with interpersonal problems than expected. Nevertheless, GoF was high (0.89) with an optimal cosine curve function with a predicted peak in the hypothesized non-assertive (HI) octant.

## 4. Discussion

The purpose of this study was to examine the psychometric properties of a brief version of the AII in a sample of patients with PD. This was tested through analyses of internal consistency of scales, standardized mean differences between short- and long-form scores, and examination of the associations between the short and long version of the AII. Furthermore, the internal structure of the instrument was assessed by CFA, and associations with different external criterion variables (i.e., alexithymia, symptom distress, interpersonal problems and personality functioning) were examined.

In sum, the results indicate that it is possible to measure AI in a reliable and valid manner using the AII-SF-42 in patients with PD. Thus, generally high internal consistency, small and negligible deviations in magnitude, very strong to perfect correlations between corresponding short- and long-form scores, a latent factor structure corresponding to the theoretical underpinnings of the construct, and the demonstration of convergent/discriminant associations with external criteria (including theoretically consistent sinusoidal patterns of relationships between AII-SF-42 affect scores and specific types of interpersonal problems) provided direct support for the psychometric soundness of the instrument and its theoretical foundations.

### 4.1. Internal domain study

Internal consistency of the AII-SF-42 scores was generally high with alphas for higher order scales in the range of 0.89–0.78. Alphas for specific affect scores were more variable, but most (five out of nine) had alphas in the range of.85–0.70. Four affect scores had alphas below.70, but all were either close to or above 0.60. Still, some subscales had alphas on the low side and future research will be needed to make sure that these scales are sufficiently reliable. Also, alphas were generally somewhat lower than those reported in the non-clinical validation of the instrument and may suggests a larger variability in the interpretation of items related to these affects in clinical and PD-samples as compared to non-clinical ones. Still, when comparing the present short-form reliabilities with those obtained using the complete 112-item version reported by Solbakken et al. (2017) and Frederiksen et al. (2022), we note, in line with Solbakken and Monsen (2021), that the reductions in reliability are arguably fairly small considering the removal of 70 items from the long version. Also, scores were generally very similar in magnitude to their full-scale counterparts. The most significant deviation was for Integration of Joy, for which the short form yielded scores.46 standard deviations higher than the long form. This difference can be categorized as small (Cohen, 1988), and we believe that the absolute differences in scores between short- and long forms can be thought of as trivial. Similarly, correlations between corresponding scores on the two versions were uniformly very high, with eight of the twelve scales having inter-correlations above 0.90 and the remaining being above.80. In sum, the inter-correlations indicated near perfect or very strong agreement in rank order of scores from the two versions of the instrument. We thus conclude that the AII-SF-42 yields reliable scores that are closely aligned to those from the long form of the instrument also in a PD-sample.

Confirmatory factor analyses demonstrated that a hybrid model specifying both an overall general AI-factor and the integration of differentiated, discrete affects was superior to competing structural conceptualizations of affect integration. This finding is in line with previous research on both short- and long forms of the AII (Solbakken et al., 2017; Solbakken and Monsen, 2021; Frederiksen et al., 2022) and findings from the similarly structured, observer-rated ACI (Solbakken et al., 2011a,b) by demonstrating the centrality of affect differentiation. At the same time, our results expand upon those previous findings by also identifying the usefulness and structural soundness of operating with a score at the overall or global affect integration level. Scores on this level have been a central part of the conceptualization of AI, but the structural inter-relationship between this superordinate score and scores at the specific affect level has never been empirically demonstrated. In sum, the AII-SF-42 ratings appear to reflect their conceptual basis well. Scores are simultaneously represented by one general overarching AI-factor and nine specific factors, each reflecting the functional integration of a discrete affect or emotional state. The obtained factor structure appears consistent with the conceptual assumptions of the underlying affect integration construct, i.e., that different affects appear to have different experiential and expressive properties and may usefully be differentiated in assessment.

### 4.2. External domain study

Overall, the examination of the predicted relationships between AII-SF-42 and related external concepts supported the concurrent, convergent, and discriminant validity of AII-SF-42 scores. Hence, scores from the AII-SF-42 were associated in systematic and expected ways with alexithymia, levels of symptom distress, levels of interpersonal problems, and various aspects of personality functioning. In same manner, the data supported the distinction between the capacity for experience and the capacity for expression by displaying theoretically consistent and differential associations with symptom severity and overall interpersonal problems. However, unlike previous studies demonstrating convergent and discriminant validity of AI-scales against expressive and experiential sub-domains of alexithymia, we could not demonstrate statistically significant differences in correlation magnitude. Still, in absolute terms, the obtained pattern of correlations was as expected and trended towards significance. Thus, we believe chance and a lack of power the most likely explanation of these non-significant findings.

Results demonstrated the hypothesized close relationship between AI and psychological distress and overall interpersonal problems. Furthermore, as expected, the levels of symptom distress and interpersonal problems were more dependent on the capacity for experiencing affects than on the capacity for expressing them. This result is in line with previous research (Monsen et al., 1996; Solbakken et al., 2011a; Frederiksen et al., 2021a; Solbakken and Monsen, 2021) and contributes to an increasingly strong case for the particular importance of experiential aspects of affective functioning for these domains.

Also, in accordance with our hypotheses, close associations were found between the AII-SF-42 scores and the SIPP-118 domains of Identity Integration, Relational Functioning and Self Control, while more modest relationships were found with the SIPP domains of Responsibility and Social Concordance. These results too support findings from previous studies (Johansen et al., 2016; Frederiksen et al., 2021a) and demonstrate that robust relationships between AI and personality functioning is reliably identified using both the conventional and short version of the AII. Bolstering previous research in the field, the results clearly point to the centrality of affective dysfunction in the constitution of maladaptive personality functioning.

In terms of patterns of convergent and discriminant relationships between the integration of specific affects and different types of interpersonal problems, results proved in accordance with hypotheses. Thus, all nine affect scores had correlation patterns that aligned with our expectations, i.e., having good fit with optimal cosine curve functions peaking in expected and separate octants of interpersonal space. The findings thus strongly support the convergent and discriminant validity of the discrete affect scores of the AII-SF-42, by demonstrating differentiated patterns of correlations systematically peaking in predicted and separate octants of the IIP-64 and low points in the corresponding, opposite octants of interpersonal space, along with good fit with respective predicted optimal cosine curve functions. Beyond supporting the construct validity of AI as assessed with the AII-42-SF, our findings provide additional support for a highly differentiated affect system and consequent importance of differentiation between affects or emotion states in assessment as proposed by, e.g., Solbakken et al. (2011a), Solbakken et al. (2017), and Frederiksen et al. (2021a).

### 4.3. Strengths and limitations

Strengths of the study are its well-characterized clinical sample, sophisticated statistical methods, and systematic data analyses, through which the totality of results constitutes an interconnected set of complementary arguments supporting the construct validity of AI as operationalized by the AII-SF-42. However, several limitations should be noted. First, a larger sample size would clearly have been preferable. Even though this issue has been considered in the analyses and interpretation of results of the CFAs, some uncertainty is still associated with estimated parameters and the subject to variable ratio was less than desirable. Still, results were conceptually meaningful, indicating that random error associated with low statistical power was not an obvious issue. Second, all instruments were self-rated, which can have inflated some associations (thus producing stronger correlations between scores than those existing in actuality). Still, the magnitudes of obtained associations are large enough that even moderate to large amounts of inflation due to common method variance probably would not change the conclusions of our study. Third, only the complete AII was administered to patients, and short- and full-form scores were computed from the same pool of data. Thus, it is possible that filling in all items together may influence item-scores on the short-form scales in ways that produce different results from filling in the short-form items only. Fourth, the cross-sectional nature of the study precludes addressing potential causality. Fifth, one should be cautious when generalizing from our findings to patients with PDs in general, since most of the sample were female and had either borderline PD or avoidant PD. Our findings therefore most clearly pertain to these two PD-categories and more clearly to females than males. The issues of sample size, the administration procedure, lack of a causal design, and uncertain representativity of the sample all point to the need for future studies to build more robust support for the validity of the AII-42-SF in samples with PDs.

## 5. Conclusion

This study is, to our knowledge, the first of its kind to examine the psychometric properties of the AII-SF-42 in a clinical sample of patients suffering from PD. Results demonstrated that AII-SF-42 scores in general have adequate internal consistency reliability and are closely aligned with corresponding scores from the longer 112-item version of the instrument in a clinical context. The factor-structure of scores conformed well to the theoretical model underlying the construct and design of the instrument. The associations between AII-SF-42 scores and various scores from external instruments indicated robust and theoretically expectable patterns of concurrent, convergent and discriminant relationships, both for the higher-order scale scores and on the level of discrete affect scores. In sum, we believe the results of the study appear convincing and deliver support for the construct validity of the AI construct as operationalized by the AII-SF-42. The present study shows that it is possible to measure the capacity for AI in a reliable and valid manner through an easily accessible and time-efficient self-report format with patients diagnosed with PDs. Results indicate that the AII-SF-42 appears to be a well-functioning alternative for those who do not have the time or space required for the application of the more comprehensive and thus time-consuming alternatives for assessing AI.

## Data availability statement

The datasets presented in this article are not readily available because no consent for data sharing was given by participants. Requests to access the datasets should be directed to ckf@rn.dk.

## Ethics statement

The study was approved by the Danish Data Protection Agency on 1 May 2014 (2019-017816). Due to the nature of the study, no further approval was needed from the Danish National Committee on Biomedical Research Ethics. The study was conducted in accordance with the local legislation and institutional requirements. The participants provided their written informed consent to participate in this study.

## Author contributions

CF, GK, and OAS: conceptualization and methodology. CF and OAS: formal analysis, interpretation of data, and writing-original draft preparation. CF, GK, and OAS: writing-review and editing. CF: project administration. All authors contributed to the article and approved the submitted version.
